# Hydrophilic and Positively Charged Polyvinylidene Fluoride Membranes for Water Treatment with Excellent Anti-Oil and Anti-Biocontamination Properties

**DOI:** 10.3390/membranes12040438

**Published:** 2022-04-18

**Authors:** Zirui Wang, Shusu Shen, Linbin Zhang, Abdessamad Ben Hida, Ganwei Zhang

**Affiliations:** 1 School of Environmental Science and Engineering, Suzhou University of Science and Technology, 1 Kerui Road, Suzhou 215009, China; 15956940790@139.com (Z.W.); lb_zhang2022@163.com (L.Z.); abdessamadbenhida@gmail.com (A.B.H.); zhangganwei214036@126.com (G.Z.); 2 Jiangsu Engineering Research Center for Separation and Purification Materials and Technology, 1 Kerui Road, Suzhou 215009, China

**Keywords:** blend membrane, water treatment, antifouling modification, hydrophilic, polycationic liquid

## Abstract

Membrane fouling limits the rapid development of membrane separations. In this study, a blend membrane containing polycationic liquid (P(BVImBr_1_-co-PEGMA_1_)) is presented that can improve the antifouling performance of polyvinylidene fluoride (PVDF) membranes. By mixing the polycationic liquid into PVDF, an improved membrane-surface hydrophilicity and enlarged membrane porosity were detected. The water contact angle decreased from 82° to 67°, the porosity enlarged from 7.22% to 89.74%, and the pure water flux improved from 0 to 631.68 L m^−2^ h^−1^. The blend membrane surfaces were found to be always positively charged at pH 3~10. By applying the membranes to the filtration of oil/water emulsion and bovine serum albumin (BSA) solution, they showed a very high rejection rate to pollutants in wastewater (99.4% to oil droplets and 85.6% to BSA). The positive membrane surface charge and the increased membrane hydrophilicity resulted in excellent antifouling performance, with the flux recovery rates of the dynamic filtration tests reaching 97.3% and 95.5%, respectively. Moreover, the blend membranes demonstrated very low BSA adhesion and could even kill *S. aureus*, showing excellent antifouling properties.

## 1. Introduction

Membrane separation technology has great potential in water and wastewater treatment. Based on the pore size diameter, membranes are often classified into microfiltration membranes, ultrafiltration membranes, nanofiltration membranes, and reverse osmosis membranes [1]. According to different membrane materials, membranes can be divided into inorganic membranes, organic membranes, or organic-inorganic hybrid membranes. Among various matrix membrane materials, polyvinylidene fluoride (PVDF) is the most-used organic polymer membrane material due to its thermal stability, excellent chemical resistance, and good membrane-forming ability [2]. 

However, PVDF is a semi-crystalline polymer with repeating units of –CH_2_–CF_2_– that form hydrophobic structures that make the membrane prone to fouling, which leads to a reduction in membrane performance by reducing permeability and imposing additional costs for membrane cleaning or replacement [3]. Membrane fouling leads to easy blockage of membrane pores, increases the transmembrane pressure, and decreases the water flux. In addition, it increases the cleaning cost of the membrane and, finally, shortens the service life of the membrane. It is generally believed that a hydrophilic membrane could somehow overcome membrane fouling problems [4,5,6,7], because the hydrophilic membrane easily forms a hydration layer on the membrane surface that can effectively reduce the adhesion between pollutants and the membrane surface. In addition, the charge on the membrane surface is also an important factor that slows down membrane fouling [8], because some pollutants in water are charged. The electrostatic action between the membrane surface and the charged pollutants in water can reduce the adhesion of pollutants, and even improve the interception effect of the membrane on charged pollutants [9,10]. 

To achieve antifouling PVDF membranes, different kinds of modifications have been developed [11,12]. The modification methods for PVDF membranes mainly include membrane surface modifications such as surface coating [13,14] and surface grafting [15,16], and membrane bulk modifications such as copolymerization modification [17,18] or blending modification [19,20]. Among them, blending modification can afford uniform membrane structure, consistent chemical composition, good separation effect, etc. 

Polyionic liquids, also known as polymer ionic liquids [21], have been reported to improve the hydrophilicity of membranes and charge the membranes, so they are quite applicable in solving the membrane fouling problem [22,23]. Many of these zwitterions have been employed as polymer brushes to modify the surface of materials with enhanced hydrophilicity and antifouling ability [24,25]. By blending a polyionic liquid, P(MMA-co-BVIm-Br) with PVDF, the obtained membranes showed a reduced fouling rate from 68% to 40% [26]. 

In our previous report [27], a polycationic liquid P(BVImBr_1_-co-PEGMA_2_), was blended with PVDF. The prepared membranes showed good repellence (up to 99%) against positively charged BSA, and the flux recovery rate was improved to 76%. However, the pollutant object of membrane treatment was limited, and the research was not deep enough. 

For our ongoing study, blend PVDF membranes with a newly synthesized polycationic liquid, P(BVImBr_1_-co-PEGMA_1_) (P11), were fabricated. The major difference in this work is that the monomer ratio of the copolymer was switched from 1/2 to 1/1. However, the membrane performance was found to be greatly improved. The properties of the modified membranes were carefully examined and the separation efficiency of different organic pollutants, including the oil droplets and the typical protein, BSA, were explored in this work. In addition, the antibacterial properties of the membrane were also tested.

## 2. Materials and Methods

### 2.1. Materials

PVDF (FR 904, >99.5%, Mw 400,000) was purchased from 3F New Materials Co. Ltd., Shanghai, China. Poly(ethylene glycol) methyl ether methacrylate (PEGMA, average Mn 950, contains 300 ppm BHT and 100 ppm MEHQ as inhibitor) was purchased from Aldrich (St. Louis, MO, USA). 1-Vinylimidazole (C_5_H_6_N_2_, 99%), 1-bromobutane (C_4_H_9_Br, >99%), N,N-dimethylformamide (DMF, AR) and azobisisobutyronitrile (AIBN, 98%), n-hexadecane (AR, 98%) were supplied by Macklin Biochemical Technology Co., Ltd., Beijing, China. BSA (Mn 67 kDa) was obtained from Aladdin Chemical Co., Ltd., Shanghai, China. Other chemicals utilized in this study were purchased with analytical quality and purified before use. Deionized (DI) water (18.2 MΩ) purified with a Milli-Q system from Millipore (Burlington, MA, USA) was used to prepare all solutions as needed in the work.

### 2.2. Synthesis of Polycationic Liquid P(BVImBr_1_-co-PEGMA_1_) (P11)

**Figure 1:**
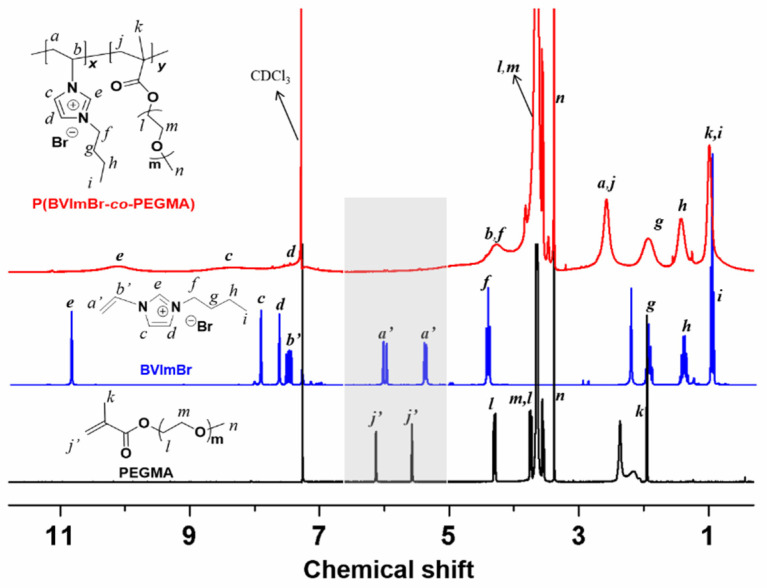
The ^1^H NMR spectra compare of the obtained P11 and the starting materials.

**Figure 2:**
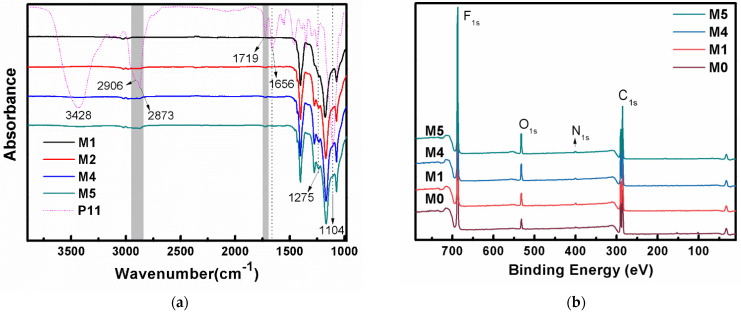

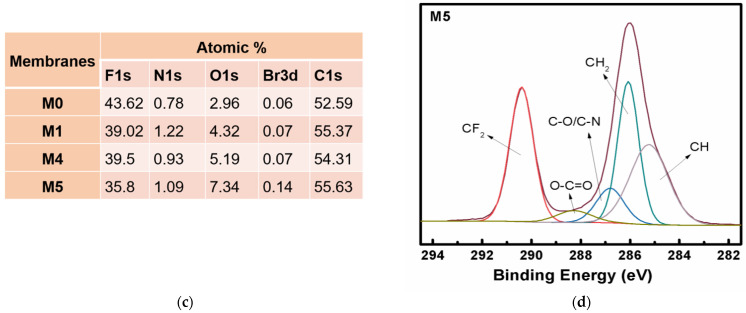
(**a**) FT-IR spectra of P11 and membranes; (**b**) full XPS spectra of membranes; (**c**) the elemental composition of membrane surface; and (**d**) the C core layer XPS spectrum of M5.

The utilized polycationic liquid, or so-called cationic polyionic liquid, (P(BVImBr_1_-*co*-PEGMA_1_)) was synthesized by following the similar protocol in our previous research [27], but in a different monomer ratio (1/1). The product is denoted as P11 in later description. It was characterized by ^1^H NMR (Bruker Avance 300) and IR analysis. Figure 1 shows the ^1^H NMR spectra (using CDCl_3_ as internal standard) of the obtained P11 and the starting materials. The disappearance of the peaks *a’* originated from BVImBr and the peaks *j’* originated from PEGMA indicates that the product P11 was successfully synthesized via the RAFT reaction, where the ratio of BVImBr/PEGMA was around 1/1. The IR analysis can be found in the latter Figure 2a.

### 2.3. Preparation of Membranes 

The flat sheet membranes were prepared by blending P11 with PVDF in different weight ratios and the polymer concentrations were 20 wt% and 22 wt%, respectively, via a non-solvent-induced phase separation (NIPS) method [27]. Six different membranes were prepared in this study; the composition of the casting solution and some of the properties are summarized in Table 1. The polymer concentrations were 20 wt% (M0, M1, M2) and 22 wt% (M3, M4, M5), and for different membrane samples, the ratio of PVDF/P11 was 10/0 (M0, M3, pure PVDF membranes), 9/1 (M1, M4) and 8/2 (M2, M5), respectively. 

### 2.4. Characterization of Membranes

#### 2.4.1. Fourier Transform Infra-Red (FTIR) Spectroscopy

FT-IR model (Nicolet 6700) was used to analyze the surface functional groups of the membranes, with a spectral range of 500~4000 cm^−1^ and a resolution of 2 cm^−1^. 

#### 2.4.2. Morphological Analysis

XPS measurement was carried out on the membrane surface using Thermo Scientific, ESCALAB 250Xi. The elements tested on the membrane surface were C, F, O, N and Br. SEM (Phenom Pro, USA) was used to observe the surface, cross-sectional structure, and thickness of membrane samples. AFM (Bruker Dimension Icon, Tucson, AZ, USA) was used to measure the surface morphology and roughness of the membranes; the sample tested was 5 μm × 5 μm.

#### 2.4.3. Contact Angles

The static water contact angle and underwater oil contact angle of the membranes were measured by the static hanging drop method, with a membrane surface contact angle tester (Ramé-Hart 500). The contact angle was measured at 5 different positions on each sample, the average value was calculated and recorded with the obtained data, and the accepted error range was less than 3.

#### 2.4.4. Mechanical Characterization

The mechanical strength was tested by a tensile strength tester (5944, Instron, Norwood, MA, USA); 3–5 strips of 5 cm × 1 cm were measured from different positions, and the average value was recorded. 

#### 2.4.5. Zeta Potential and Thermogravimetric Analysis

The membrane surface charge properties were measured with a membrane solid sample flow field potential analyzer (Surpass 3). An SDT 2960 analyzer was used for thermogravimetric analysis (TGA).

#### 2.4.6. Porosity and Pore Size

Aperture measurement involved randomly measuring 100 membrane pores on the surface image of electron micrograph with Nano Measure, and the average pore size D (nm) was calculated. The membrane porosity was measured by cutting each membrane sample into circular slices with a diameter of 2.5 cm, which were washed in ethanol and then soaked in deionized water for 24 h. The soaked membranes were removed and residual water on the membrane surface was gently wiped with dust-free paper. The mass of the wet sample was weighed and recorded as m_1_. The wet membranes were dried in a vacuum drying oven (VD115, BINDER, Tuttlingen, Germany) at 60 °C until a consistent mass was obtained, m_2_. The membrane porosity ε was calculated by Equation (1):

(1) $$\varepsilon =\frac{m_{1}-m_{2}}{\rho \cdot A\cdot d}\times 100\%$$

where ε represents the porosity (%), m_1_ and m_2_ are the wet weight and dry weight (kg), ρ represents the density of water (kg m^−2^), A is the area (m^2^), and d is the thickness (m) of the membrane.

### 2.5. Membrane Performance: Pure Water Flux and Dynamic Filtration Tests

A pure water flux test was carried out by using a dead-end filtration system. The membrane sample was cut into a certain size wafer and fixed in an ultrafiltration cup, with an effective filtration area of 8.55 cm^2^. At first, the membrane was pretreated under 0.12 MPa pressure for 30 min, and was then filtered by pure water under 0.1 MPa pressure. The filtered pure water was collected every 10 min and the volume (L) of the collected water was measured and recorded. Until the effluent volume reached a stable value, the pure water flux J_0_ was calculated by Equation (2):

(2) $${\;J}_{0}=\frac{V}{S\cdot t}$$

where J_0_ is pure water flux (L m^−^^2^ h^−1^), V is the volume of pure water passing through the membrane (L), S is the effective area of water passing through (m_2_), and t is the time of each water intake (h).

Oil/water emulsion separation: The oil/water emulsion (0.1 g L^−1^) was prepared by using 0.5 g of n-hexadecane diluted into 5 L deionized water. The operation for the oil/water separation was carried out by following the pure water flux test. After the stable flux J_0_ of pure water was calculated, the oil/water emulsion was continuously filtered by the same membrane sample for another 120 min. The flux of oil/water emulsions for 1 min was recorded every 10 min. The permeation flux after 120 min was recorded as J_p_. Then, the fouled membrane was taken out and placed in deionized water for 10 min for ultrasonic cleaning. The cleaned membrane was put back into the dead-end filtration system for testing with another pure water flux and the data were recorded as J_c_. During the filtration of oil/water emulsion, the oil concentration was detected with an organic carbon analyzer (TOC-L CPH, Shimadzu, Japan). The dynamic antifouling properties were evaluated by the flux decay rate (RFD) and the relative flux recovery rate (RFR) according to Equations (3) and (4) below:

(3) $$\mathrm{RFD}=\frac{J_{c}}{J_{0}}\times 100\%$$

(4) $$\mathrm{RFR}=\left(1-\frac{J_{p}}{J_{0}}\right)\times 100\%$$

where J_0_ is the initial pure water flux (L m^−^^2^ h^−1^), J_p_ is the permeation flux (L m^−^^2^ h^−1^) after running for 120 min, and J_c_ is the pure water flux (L m^−^^2^ h^−1^) measured after membrane cleaning.

BSA solution separation: 1.0 g L^−1^ of BSA solutions at different pH were prepared by using phosphate buffer or acetic acid/sodium acetate buffer. The filtration operation of the BSA solution was almost the same as the oil/water emulsion separations. The concentrations of BSA before and after the filtration were measured by a UV-vis spectrometer. The rejection rate (R) was obtained by Equation(5):

(5) $$R=\left(1-\frac{C_{p}}{C_{r}}\right)\times 100\%$$

where C_p_ represents pollutant concentration in the filtrate (mg L^−1^) and C_r_ represents pollutant concentration in feed liquid (mg L^−1^). 

### 2.6. Static Antifouling Tests

The BSA adsorption experiment proceeded as follows: 0.1 g of dried membrane sample was placed into 50 mL of 1.0 g L^−1^ BSA solution (pH 7.0) and shaken for 24 h. After that, the membrane was taken out of the solution and the BSA concentration of the residual solution was measured by UV-vis spectrometer. The adsorption capacity (mg g^−1^) of the membrane was then calculated.

The antibacterial test of the membranes was carried out via *S. aureus* suspension by first cutting the sterilized membrane sample into a circular piece with a diameter of 15 mm and laying it on the bottom of the 24-well plate. 100 µL of *S. aureus* suspension (10^6^ CFU mL^−^^1^) was extracted and dropped onto the membrane sample, and the 24-well plate was placed in the shaking table at 37 °C and 80 rpm. After incubation for 2 h, the bacterial solution was resuspended with 900 µL PBS. Then, 100 µL of bacterial solution after resuspension was extracted and spread evenly onto a solid medium. The coated solid medium was put in the shaking table and incubated upside-down for 24 h at 37 °C. Finally, the bacterial culture dish was photographed and the number of the remaining *S. aureus* was counted using Image J software. Each experiment was repeated at least three times.

## 3. Results and Discussion

### 3.1. Surface Chemical Composition of Membranes

FT-IR analysis was conducted to determine the surface chemical composition of the membranes. The IR spectrum of P11 is also shown in Figure 2a, with the peaks at 1104 cm^−1^ and 1275 cm^−1^ attributed to stretching vibration and bending vibration of the C-O bond, and the peak at 1719 cm^−1^ assigned to the C=O double bond in the PEGMA segment. In addition, 1656 cm^−1^ can be assigned to C=C and C=N double bonds in the BVIm-Br segment, and the peaks at 2873 and 2906 cm^−1^ must be the saturated C-H bonds that present in P11. Compared with pure PVDF membrane M0, the blend membranes M1, M4, and M5 showed obvious characteristic peaks at 1719 cm^−1^ and 2873~2906 cm^−1^, with the peak intensity enhanced with increased blending ratios of P11. 

XPS analysis of the membrane surface further proved the successful blending of P11. Figure 2b shows the full XPS spectra of the pure PVDF membrane (M0) and the blend membranes M1, M4, and M5. Compared with M0, the blend membranes containing P11 had a new peak of N1s at 401 eV, and the peak area enhanced with the increasing content of P11. The peak area of O1s at 532 eV also increased due to the greater content of P11 in the polymer mixture. The elemental composition of the membrane surface is shown in Figure 2c; by increasing P11 in the membrane from M0 to M5, the atomic ratio of F atom on the membrane surface decreased and the atomic ratio of N, O, and Br atoms improved gradually. Figure 2d is the C core layer XPS spectrum of the blend membrane M5; the peak analysis indicated characteristic peaks at 288 eV and 287 eV, which can be corresponded to O-C=O and C-O/C-N bonds which exist in the blended P11. 

### 3.2. SEM Images of the Membranes 

**Figure 3:**
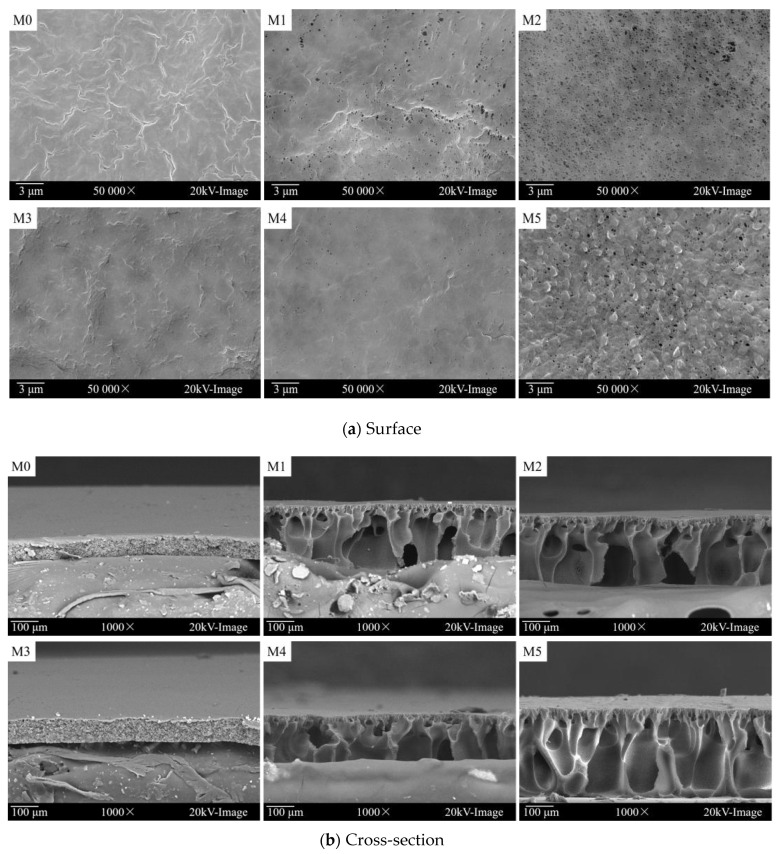
The SEM images of all membranes. (**a**) Surface; (**b**) Cross-section.

As shown in Figure 3a, no obvious pore can be found on the surface of pure PVDF membranes (M0 or M3). Comparably, more pores appeared on the blend membrane surface. The porosity data are summarized in Table 1; for example, the porosity of the pure PVDF membrane M0 was 7.21%, and the porosity of blend membrane M2 became 89.24%, almost 13 times more than M0. This is mainly due to the hydrophilic polyionic liquid (P11) that may accelerate the mass transfer rate of the solvent and non-solvent during the phase transfer stage, thus accelerating the transient liquid–liquid phase separation [28], producing a more porous and loose membrane structure. It can be seen from the cross-sectional view (Figure 3b) that the blend membranes (M1, M2, M4, M5) all showed typical asymmetric structure. With an increased ratio of the polycationic liquid mixed into the membrane, the membrane became thicker. As summarized in Table 1, the thicknesses of membranes M0, M1, and M2 were 30.4 μm, 113.6 μm and 121.2 μm, respectively. This accords with the observation of a more loose structure. In addition, by adding more P11 into the membrane, more finger-like macropores can be observed on the cross-section of the membranes.

The mean pore size of the membranes are summarized in Table 1; the pore size and the pure water flux were enlarged. For example, the blend membrane M2 gave the largest mean pore size (35.2 nm) and water flux (631.68 L m^−^^2^ h^−1^). By increasing the polymer concentration from 20 wt% to 22 wt%, the obtained membranes became denser, and the pore size or the pure water flux decreased (such as for membrane M5, reduced to 27.32 nm and 238.63 L m^−^^2^ h^−1^, respectively). 

As detected from the SEM images, the thickness of membrane M5 was the thickest (Table 1, M5: 149.0 μm); this is due to the higher polymer concentration of the casting solution and the higher ratio of the copolymer P11 (2/8 of P11/PVDF) in the membrane. Correspondingly, the mechanical strength was found to be stronger (Table 1, M5: 0.54 MPa) than membrane M2, which has a lower polymer concentration (Table 1, M2: 0.48 MPa). Although the blend membranes’ mechanical strength was smaller than that of the pure PVDF membranes (Table 1, M0: 1.82 MPa; M3: 2.01 MPa), they can survive under ultrafiltration pressure, which is usually lower than 0.30 MPa. 

### 3.3. Surface Wettability of the Membranes

**Figure 4:**
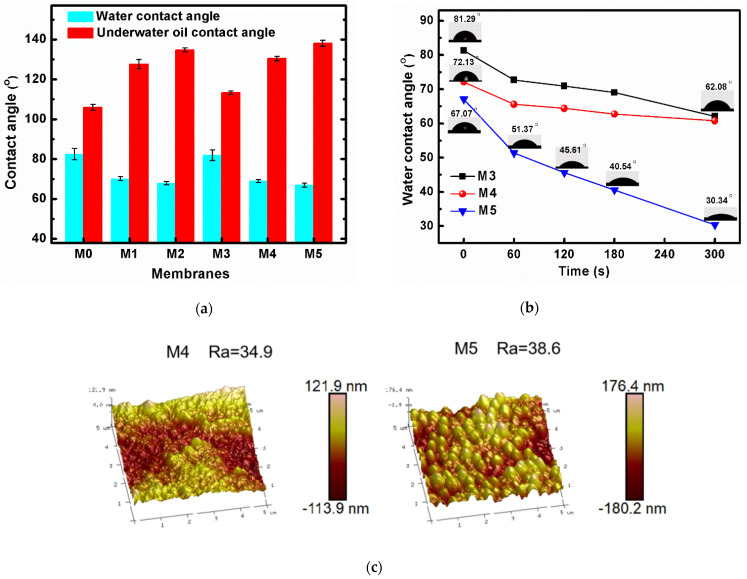

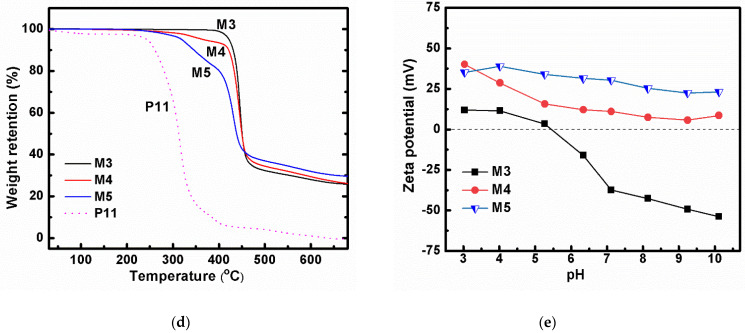
(**a**) The static water/underwater oil contact angles of all membranes; (**b**) the dynamic water contact angles of membranes M3, M4, and M5; (**c**) AFM images of blend membranes M4 and M5; (**d**) TGA diagram of the polymer P11 and membranes; and (**e**) Zeta potentials of membranes M3, M4, and M5.

The water contact angles and underwater oil contact angles of the membranes can be found in Figure 4a. The hydrophilicity and the oleophobicity of the PVDF membranes were improved by adding the polycationic liquid P11. For example, the water contact angle of the pure PVDF membrane M3 was 81.9°, its under-water oil contact angle was 113.3°, and the same data points for the blend membrane M5 were 67.0° and 138.1°, respectively. This is mainly caused by the introduction of polycationic liquid, P11, which is both hydrophilic and positively charged. The PEGMA segments in P11 helped the hydrophilicity; meanwhile, the cationic liquid (BVImBr) segments may improve both the positive charge and hydrophilicity.

The dynamic changes in water contact angles of the membranes M3, M4, and M5 are also recorded in Figure 4b, which can reflect the surface wettability of the membrane more intuitively, and the faster the wetting speed, the better the surface wettability. With the increase of immersion time, the contact angles of all membranes decreased. Compared with pure membrane M3 and blend membrane M4, the wetting speed of blend membrane M5 was much faster: the water contact angle dropped from 67.07° to 30.34° in 300 s. This is mainly because membrane M5 has a higher blending of hydrophilic P11 in the membrane. 

In addition, the AFM images (Figure 4c) show that the surface roughness of M5 was higher than M4, where the average roughness (Ra) for M4 was 34.9 nm and the Ra of M5 was 38.6 nm. There are many protrusions on the surface of the membrane M5; this phenomenon can also be found in the surface SEM images of M5 (Figure 4a, M5) as more pores appear on the surface face. Therefore, the rougher and more porous surface accelerated the membrane wetting speed. 

### 3.4. Thermal Stability and Zeta Potentials of the Membranes

TGA curves of blend membranes and the copolymer P11 are described in Figure 4d. The copolymer P11 showed obvious weight loss at 250 °C, which indicated the decomposition of the polymer. The pure PVDF membrane M3 showed the only thermal weight loss at 414 °C because the pure membrane had strong thermal stability. Compared with M3, the blend membranes M4 and M5 gave the first obvious weight loss at a temperate 250 °C, which can be attributed to the decomposition of P11; M5 gave more weight loss than M4 because M5 was fabricated from a higher content of P11 in the polymer mixture.

Figure 4e shows that by blending cationic P11 into the PVDF membrane, the membranes M4 and M5 were always positively charged at pH 3~10, while the isoelectric point of pure PVDF membrane M3 is 5.4. Moreover, with the higher content of P11, the membrane M5 (2/8) appeared to be more positively charged than M4 (1/9). This enrichment of positive electricity on the membrane surface may help to enhance the electrostatic interaction between the membrane surface and pollutants in wastewater, which is quite beneficial for antifouling performance and the interception effects of the membrane on some charged pollutants.

### 3.5. Filtration of Oil/Water Emulsions

**Figure 5:**
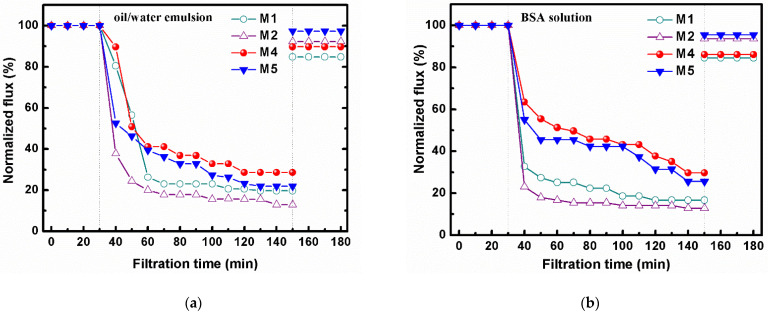
The normalized flux during the filtration of: (**a**) oil/water emulsions (0.1 g L^−1^, pH 7) and (**b**) BSA solutions (1.0 g L^−1^, pH 3.6).

Figure 5a and Table 2 describe the filtration performance of blend membranes M1, M2, M4, and M5 in separating 0.1 g L^−1^ of oil/water emulsion. All the membranes showed a rapid flux decay within 30~60 min, which was attributed to the fast aggregation of oil droplets on the membrane surface and even blockage of internal membrane pores driven by pressure. Rates of higher than 91% in oil rejection were obtained for all the membranes, with membrane M2 giving the lowest R at 91.6% and membrane M4 showing the highest R (up to 99.4%). This indicates that the rejection mechanism of the membranes is mainly due to the pore size screening effect. It was mentioned in a previous section that M4 had the smallest pore size and M2 had the largest pore size; the data are shown in Table 1. 

Despite the high flux decay, the blend membranes demonstrated good antifouling properties after an ultrasonic cleaning by DI water; up to 97.3% of RFR can be found for membrane M5. The RFR for M1 was 84.8%; by adding more polycationic liquid P11 into the membrane, a better RFR was obtained. This can be explained by the fact that on a more hydrophilic membrane surface, a denser hydrated layer can be formed, which can effectively prevent the adhesion of oil droplets. 

The R and RFR of membranes M1, M2, M4, and M5 in the repeated filtration cycles of oil/water emulsion are summarized in Table 2. Both the flux recovery and the rejection rate of each membrane sample decline after three repeated filtrations. For example, the rejection rate of M5 in each cycle were 98.5%, 94.1%, and 87.3%. The decrease in the membrane rejection may be caused by the clean method utilized in this experiment, such that the ultrasonic clean might damage the membrane pores and therefore affecting the pore size screening effect. Although the RFR was also reduced in each cycle, membrane M5 showed the smallest decline while the RFR of M5 in the third cycle remained as high as 89%.

### 3.6. Filtration of BSA Solutions

A similar operation was employed in the filtration of BSA solutions (Figure 5b, 1.0 g L^−1^, pH 3.6). During the BSA separation, the changing trend was quite similar to the oil/water emulsion separation (Figure 5a). The blend membranes M4 and M5 showed reduced flux decay (Table 3, 70.3% and 74.4%) as compared to membranes M1 and M2 (Table 3, 83.4% and 87.1%); M5 gave the best relative flux recovery rate (RFR) at 95.5%, indicating the improved antifouling property. The explanation follows the previous discussion on the oil/water separation. As shown in Table 3, the rejection rates of M4 and M5 were much higher than that of M1 and M2 due to the decreased membrane pore size.

By using the optimum membranes M4 and M5, which have narrower pore sizes and good BSA rejections, three repeated filtration cycles were carried out to evaluate the membrane performance. As demonstrated in Table 3, despite the slight decreased R and RFR in every cycle, the RFR of M5 was still as high as 88.3%, with the blend membranes with the highest content of P11 showing good anti-protein-fouling properties.

It is noted that the pH value of the BSA solution was 3.6 and the isoelectric point of BSA is 4.7, and thus, the BSA molecules at pH 3.6 appears to be positively charged. The membranes M4 and M5 have been confirmed to have positively charged surfaces: the electrostatic repulsion between the positive BSA and the positive membrane is suggested to affect the pollutant rejection and the enhanced antifouling performance. 

A test of BSA rejection at different levels of pH was carried out by using the membrane M5, which possessed the higher ratio of P11 in the polymer mixture, but in different polymer concentrations. Rejections of M5 were 85.6% (pH 3.6), 81.5% (pH 4.7) and 86.4% (pH 7.0), respectively. Given the lowest rejection of BSA at pH 4.7 and the fact that the BSA molecules were neutral and so no specific electrostatic interaction between BSA and membrane presented there, the pore size screening effect is the major rejection mechanism. Comparably, membrane M5 showed higher rejections when the pH values of the BSA solution were 3.6 and 7.0. They are almost the same, but higher than the neutral conditions. It proved that the retention of pollutants can be assisted by the electrostatic effects, and that both electrostatic repulsion and electrostatic attraction between the charged pollutant and the charged membrane surface help the retention improvement. 

The data of our previous work [27]—in which the different polycationic liquid P(BVImBr_1_-co-PEGMA_2_) was used in a 1/2 ratio between the two monomers and the obtained PVDF membranes were also applied into the BSA filtration—are summarized in Table 4. As mentioned at the very beginning of this report, we newly synthesized P11 and the monomer ratio was changed to 1/1. It can be seen in Table 4 that the pure water flux of the membranes were similar and the membrane hydrophilicity enhanced gradually. All of them showed ~99% BSA rejection; however, the RFR was greatly improved from 76.2% to 97.3%. This is mainly due to the increased monomer ratio of cationic liquid (BVImBr) in the synthesized polymer, which enhanced the positive charge of the membrane, and due to the higher content of cationic P11 in the polymer mixture, the membranes became more positively charged and hydrophilic, which finally helped the antifouling ability.

### 3.7. The Static Antifouling Performance

Since the blend membranes demonstrated good antifouling performance in the dynamic filtrations of typical organic pollutants (oil and protein), the static antipollution of the membranes was also evaluated by the adsorption test of BSA [29] and antibacterial tests.

Pure PVDF membrane M3 showed no adsorption capacity of BSA molecule (0 mg g^−1^), the blend membranes showed an increased adsorption capacity from M4 (0.013 mg g^−1^) to M5 (0.017 mg g^−1^). The main reason should be the increased membrane porosity that may increase the specific surface area and adsorption sites of the membranes. The electrostatic interaction between the positively charged surface of the blend membranes and negatively charged BSA (at pH 7.0) was also considered to facilitate the adsorption process, but should not be the dominant reason since the adsorption capacity data is very small. Such low adsorption ability indicated good anti-protein adhesion performance of the blend membranes, and this is mainly attributed to the good hydrophilicity.

**Figure 6:**
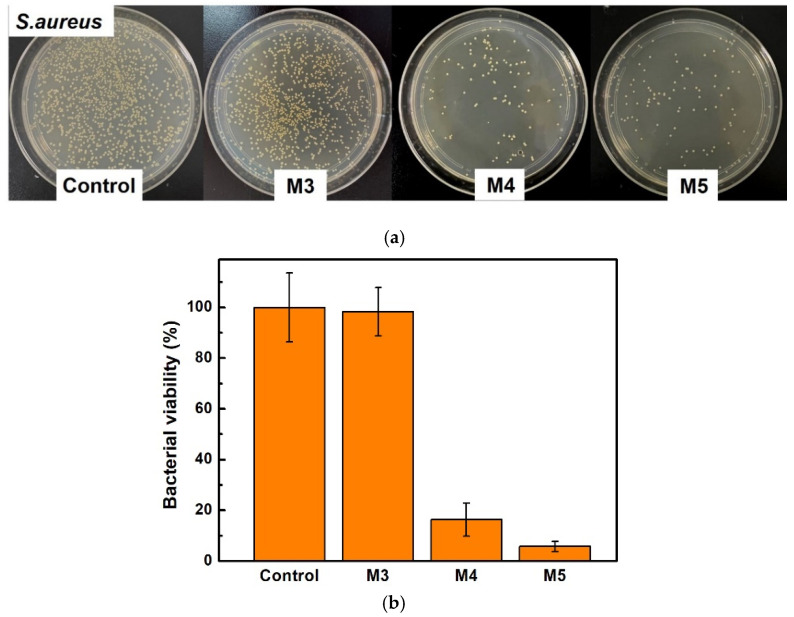
(**a**) Photographs and (**b**) the bacterial viabilities of the membranes M3, M4, and M5 against *S. aureus*.

The membranes M3, M4, and M5 were tested for the antibacterial activity against *S. aureus*. The results in Figure 6 showed the pure PVDF membrane M3 had almost no antibacterial ability, while the blend membranes M4 and M5 showed good bactericidal ability. This is due to the blending of polycationic liquid P11 in the membranes, which can interact with the negatively charged cell membrane of the bacteria, destroying the structure of bacteria and killing them [30,31]. With the increase of polycationic liquid, the number of bacteria (Figure 6a) and the bacterial activity (Figure 6b) were greatly reduced. For example, the bacterial viability of M5 was as low as 5.74%: the blend membranes showed excellent bactericidal ability. 

## 4. Conclusions

In this study, a positively charged and hydrophilic PVDF membrane has been well-developed by blending with a novel synthetic polycationic liquid, P11, via the NIPS method. By using the membranes M4 and M5, fabricated from 22 wt% of polymer concentration in the casting solution, the blend PVDF membranes showed excellent antifouling properties due to the cationic structure of and the hydrophilic segments in P11. The pure water flux has been improved to 238.63 L m^−^^2^ h^−1^ because of the increased membrane pores. During the filtration of oil/water emulsion, the membranes showed a very high R at 99.4%, and up to 97.3% of RFR was achieved after the ultrasonic cleaning of the fouled membranes. When applied to BSA solution separation, 87.2% R and 95.5% RFR can be detected for the membranes. After three repeated filtration cycles, although the rejection rates and the relative flux recovery rate declined slightly, the blend membranes still exhibited good separation and antifouling effects. Moreover, the static antifouling ability of the membranes was so strong that extremely low BSA adsorption capacity (0.013 mg g^−1^) was found, and the bacterial viability of *S. aureus* was reduced greatly from 98.9% to 5.74%. In summary, an efficient anti-pollution separation membrane has been developed which has good application prospects for water and wastewater treatment.

## Figures and Tables

**Table 1:** The casting solution composition and general properties of the membranes.

Membranes	M0	M1	M2	M3	M4	M5
Polymer mixture	PVDF	PVDF/P11 = 9/1	PVDF/P11 = 8/2	PVDF	PVDF/P11 = 9/1	PVDF/P11 = 8/2
Polymer concentration/wt%	20	20	20	22	22	22
Porosity/%	7.22 ± 1.75	75.31 ± 1.24	89.74 ± 2.01	5.18 ± 0.95	70.33 ± 1.56	82.65 ± 2.20
Mean pore size/nm	/	29.91 ± 1.02	35.20 ± 1.31	/	24.75 ± 1.14	27.32 ± 1.57
Pure water flux/L m^−^^2^ h−^1^	/	427.47 ± 10.32	631.68 ± 15.79	/	193.24 ± 7.39	238.63 ± 6.50
Thickness/μm	30.4 ± 0.28	113.6 ± 1.08	121.2 ± 0.92	39.2 ± 0.83	92.3 ± 1.02	149.0 ± 0.71
Mechanical strength/MPa	1.82 ± 0.24	0.83 ± 0.36	0.48 ± 0.15	2.01 ± 0.69	0.94 ± 0.34	0.54 ± 0.28

**Table 2:** Parameters in the filtration of oil/water emulsions.

Membranes	J_0_ /L m^−^^2^ h^−1^	J_p_ /L m^−^^2^ h^−1^	J_c_ /L m^−^^2^ h^−1^	RFD /%	RFR /%	R /%	RFR/%	R/%	RFR/%	R/%
							2nd Cycle		3rd Cycle	
M1	427.47	84.18	362.35	80.3	84.8	96.4	77.3	93.2	61.4	85.7
M2	631.68	81.49	582.41	87.1	92.2	91.6	87.8	87.4	83.4	79.6
M4	193.24	51.21	173.34	73.5	89.7	99.4	82.6	96.5	73.2	88.2
M5	238.63	50.35	232.19	78.9	97.3	98.5	92.4	94.1	88.7	87.3

**Table 3:** Parameters in the filtration of BSA solutions.

Membranes	J_0_ /L m^−^^2^ h^−1^	J_p_ /L m^−^^2^ h^−1^	J_c_ /L m^−^^2^ h^−1^	RFD /%	RFR /%	R /%	RFR/%	R/%	RFR/%	R/%
							2nd Cycle		3rd Cycle	
M1	439.47	73.15	371.45	83.4	84.5	38.4		not tested		
M2	547.45	70.19	512.36	87.1	93.6	31.0				
M4	186.24	55.27	160.35	70.3	86.1	87.2	82.9	79.3	76.3	65.6
M5	220.47	56.34	210.45	74.4	95.5	85.6	93.2	77.9	88.3	62.4

**Table 4:** Parameters of different membranes during the filtration of BSA solution (pH 3.6).

Membranes	Pure Water Flux	Water Contact Angle	R	RFR	Ref.
Optimum membrane in previous work: P(BVImBr_1_-co-PEGMA_2_)/PVDF = 6/15	200.8 ± 9.0 L m^−^^2^ h^−1^	76.3°	99.1%	76.2%	[27]
M4: P(BVImBr_1_-co-PEGMA_1_)/PVDF = 1/9	193.24 ± 7.39 L m^−^^2^ h^−1^	72.1°	99.4%	89.7%	this work
M5: P(BVImBr_1_-co-PEGMA_1_)/PVDF = 2/8	238.63 ± 6.50 L m^−^^2^ h^−1^	67.0°	98.5%	97.3%	this work

## Data Availability

Not applicable.
